# Exploring the experiences of Haitians/Haitian Americans in Miami-Dade County, Florida during the COVID-19 pandemic: how this community coped with the public health emergency

**DOI:** 10.3389/fpubh.2024.1364260

**Published:** 2024-08-15

**Authors:** Candice A. Sternberg, Danelle Richard, Valerie E. Daniel, Maurice J. Chery, Maika Beauvoir, Dora Marcelin, Aline Francois, Micaelle Titus, April Mann, Maria L. Alcaide, Sannisha K. Dale

**Affiliations:** ^1^Department of Medicine, University of Miami Miller School of Medicine, Miami, FL, United States; ^2^Department of Psychology, University of Miami, Coral Gables, FL, United States; ^3^Department of Public Health Sciences, University of Miami Miller School of Medicine, Miami, FL, United States; ^4^Department of Obstetrics and Gynecology, University of Miami, Miami, FL, United States; ^5^Family Action Network Movement, Miami, FL, United States; ^6^Community Health and Empowerment Network, Miami, FL, United States; ^7^College of Arts and Sciences, University of Miami, Coral Gables, FL, United States

**Keywords:** COVID-19, Haitians, Haitian Americans, healthcare, Miami-Dade, pandemic

## Abstract

**Objective:**

To explore and describe the experiences of Haitians/Haitian Americans in Miami-Dade County, Florida during the COVID-19 pandemic, including their attitudes and practices towards vaccination.

**Design:**

We interviewed 15 community members and 15 stakeholders in the Haitian/Haitian American community in Miami-Dade County, Florida using a semi-structured interview guide. The qualitative interviews were conducted between February 4, 2021, and October 1, 2021. They were conducted in both English and Haitian Creole, audio recorded transcribed/translated, and coded using thematic content analysis.

**Results:**

The analyses revealed 9 major themes: (1) thoughts about the pandemic, (2) concerns about the COVID-19 vaccines, (3) healthcare access, February–October 2021, (4) intrapersonal relationship dynamics, (5) thoughts about individuals diagnosed with COVID-19, (6) thoughts about prevention measures (e.g., wearing masks, hand hygiene, social distancing, vaccination), (7) mental health struggles and coping, (8) food insecurity, and (9) overall experiences of the pandemic. The findings reveal that the COVID-19 public health emergency negatively affected Haitians/Haitian Americans across several domains, including employment, healthcare access, personal relationships, and food security.

**Conclusion:**

This research echoes the compounding negative experiences reported by multiple disadvantaged groups during the COVID-19 pandemic. From loss of employment to healthcare barriers, the pandemic forced many Haitians/Haitian Americans into greater economic and social instability. Interventions addressing these issues should recognize how these factors may interact and compound the experiences of this group. Health and public health agencies should work alongside community partners to build trust so that preventive efforts will be more readily accepted during public health emergencies.

## Introduction

The first documented COVID-19 death in Miami-Dade County was a 94-year-old Haitian woman, and from that point on, Haitians/Haitian Americans were severely impacted by the pandemic (1). Although COVID-19 negatively affected the entire world, within the United States (U.S.) and Florida (FL), its impact on racial and ethnic minorities was devastating, with those populations having higher than average rates of infection, hospitalization, and mortality (2). Since the pandemic began in 2020, African American/Black individuals have consistently faced a disproportionate burden of COVID-19-related health and socioeconomic risks (3). Unfortunately, state health officials did not always routinely track diagnoses by ethnicity, and Haitian/Haitian Americans, like other Afro-Caribbeans, are often grouped with African Americans in reporting, confounding attempts to parse the experiences of different ethnic groups (1, 4). Haitians reportedly account for about 4% of the population in Miami-Dade County (1). Miami-Dade County has a population of approximately 2.7 million individuals (5). Haitian Americans accounted for at least 5% of those affected by COVID-19. As of the end of August 2020, at least 105 of the more than 2,000 deaths in Miami-Dade County had been members of the Haitian community. This is thought to be an under count (1). Given missing ethnicity data for hospitalizations, it is not possible to discern if Haitians were disproportionately affected (1). Many factors contributed to the disproportionate negative effects of COVID-19 on Black individuals, including linked historical structural inequities (e.g., racism, poverty, food deserts), more crowded and polluted living conditions, a higher likelihood of having public front-facing employment (e.g., service, food, transportation industries), and the population’s likelihood of having more pre-existing comorbidities (e.g., diabetes, cardiovascular disease) (6). Economic instability likely amplified the pandemic’s negative consequences for many Haitians and Haitian Americans (4). The Haitian community is an ethno-racial, linguistic community which also contains individuals with low income. Data from the Thrive305 survey conducted in Miami-Dade County by the government collected data from participants of Haitian descent during the COVID-19 pandemic indicated evidence of discrimination. When answering the question, I think that residents of all backgrounds including my own, are welcomed and respected “by fellow residents of Miami-Dade”, the highest numbers of responses were “disagree.” In addition, when asked “I think that residents of all backgrounds, including my own, have equal employment opportunities in Miami-Dade County,” the highest numbers of responses were “strongly disagree” (7).

The limited current literature suggests that the negative effects of the COVID-19 pandemic were felt disproportionately by Haitian/Haitian Americans in many ways (2, 4). For example, 40% of the Haitian diaspora in the U.S. works in public service-related industries, which were among the first to face major economic loss and unemployment due to social distancing policies (8). In addition, food insecurity for many increased, and Black and Latino households were twice as likely as white households to report that their families did not have enough to eat (9). COVID-19 hurt social relationships and mental health as well (9). In a case study conducted in Miami Gardens, found that Black community members rated their physical and mental health less positively since the pandemic began, reporting increased loneliness (9, 10).

Structural interventions are needed to reduce the pandemic’s harm on this population, including making testing and vaccines free and accessible, improving tracing/reporting data, and continuing economic relief for those affected. In addition, existing prevention methods (such as wearing masks) must be better utilized to reduce morbidity and mortality. Furthermore, although vaccination is known to be the most effective mechanism to prevent COVID morbidity and mortality, vaccination rates remain low among Black communities in the U.S. and in Haiti (10, 11). Chery et al. (12) found that among 1,071 survey respondents in Haiti, only 27% had positive attitudes towards the COVID-19 vaccine and had been vaccinated.

This study aimed to better understand the experiences of Haitians/Haitian Americans, during the COVID-19 pandemic, particularly with regard to (1) thoughts/concerns about the pandemic, (2) thoughts about COVID-19 vaccines, (3) healthcare access during the study period, (4) intrapersonal relationship dynamics, (5) thoughts about individuals diagnosed with the COVID-19, (6) prevention measures (e.g., wearing masks), (7) mental health struggles and coping, (8) food insecurity, and (9) overall experiences. A better understanding of Haitians/Haitian Americans’ experiences during the COVID-19 pandemic will allow us to provide guidance on addressing its continued negative consequences and develop more effective mitigation strategies for future pandemics and other public health emergencies. The Health Equity Impact Assessment highlights social determinants of health that are relevant to Haitians/Haitian Americans and COVID-19 (13). The social determinants of health addressed in this manuscript are income/social status, social support networks, employment, social environments, personal health practices, coping skills, health services, and culture.

## Materials and methods

A qualitative research design was used to collect and analyze data. In partnership with the Family Action Network Movement and Community Health and Empowerment Network, 30 Haitians/Haitian Americans were recruited face-to-face and by phone. Participants included community members and those in community leadership positions (stakeholders) in Miami, FL. The participants did not have a relationship with the study team prior to the study. The study used convenience sampling. Data were collected between February 4, and October 1, 2021, at community-based organizations, churches, and via phone. Participants were recruited using flyers and face-to-face by study team and staff members of community partners at the Family Action Network Movement and Community Health and Empowerment Network, as well as other community-based organizations and churches across Miami-Dade County. Participants who expressed interest and met eligibility criteria were enrolled. The research team consisted of female and male staff experienced in qualitative research and bilingual in Haitian Creole and English. The interview guides were reviewed and modified with feedback from the Family Action Network Movement and Community Health and Empowerment Network. Thirty sociodemographic surveys and semi-structured interviews were conducted with Haitians/Haitian Americans (15 community members and 15 stakeholders). One potential participant was unable to complete the study due to scheduling conflicts. The interviews were conducted by the principal investigator (an MD), a research associate, a community consultant, and three other research team members in person at a private space and/or by the phone. The study team verbally read the consent form to participants and provided a copy of the document in their preferred language. All participants provided verbal informed consent to participate in this study. Interviews were audio recorded and lasted for 30–45 min, during which notes were taken. No interviews were repeated. Interviews conducted in Haitian Creole were later transcribed and translated to English. All participants were given $30 gift cards for their participation. The University of Miami Institutional Review Board (Study #: 20200190) approved this study’s protocol and materials prior to data collection in the field.

### Measures

#### Sociodemographic assessments

The verbally administered sociodemographic survey collected information on gender, age, birthplace, education, religion, housing status, income, and work/school status. Demographic characteristics can be found in Table 1. All responses were recorded into Research Electronic Data Capture (REDCap), a secure web-based application used for data collection (14).

**Table 1:** Demographic characteristics of participants (*n* = 30).

Variables	Total participants	Community members	Stakeholders
	*N* = 30	*N* = 15	*N* = 15
Gender, *N* (%)			
Male	14 (46.7%)	8 (53.3%)	6 (40.0%)
Female	15 (50.0%)	7 (46.7%)	8 (53.3%)
I choose not to answer	1 (3.3%)	0 (0.0%)	1 (6.7%)
Age, Mean (SD)			
	43.7 (13.00)	47.9 (14.27)	39.5 (10.47)
Birthplace, *N* (%)			
USA	6 (19.4%)	0 (0.0%)	6 (40.0%)
Haiti	23 (74.2%)	15 (100%)	8 (53.3%)
Other	1 (6.4%)	0 (0.0%)	1 (6.7%)
Education, *N* (%)			
Some or no high school	9 (30.0%)	9 (60.0%)	0 (0.0%)
High school graduate or GED	3 (10.0%)	2 (13.3%)	1 (6.7%)
Some College	3 (10.0%)	1 (6.7%)	2 (13.3%)
College Graduate	9 (30.0%)	2 (13.3%)	7 (46.7)
Graduate School	5 (16.7%)	1 (6.7%)	4 (26.7)
I choose not to answer	1 (3.3%)	0 (0.0%)	1 (6.7%)
Religion, *N* (%)			
Christian (Catholic, Baptist, Protestant, Non-denominational)	26 (86.7%)	15 (100%)	11 (73.3%)
None	4 (13.3%)	0 (0.0%)	4 (26.7%)
Housing arrangement, *N* (%)			
Renting a house or apartment	20 (66.7%)	10 (66.7%)	10 (66.7%)
Living in a house or apartment owned by you or someone in your household	8 (26.7%)	4 (26.7%)	4 (26.7%)
Other (temporary/transitional stay)	2 (6.6%)	1 (6.7%)	1 (6.7%)
Household income, *N* (%)			
Less than $5,000	4 (13.3%)	1 (6.7%)	3 (20.0%)
$5,000–$24,999	9 (30.0%)	7 (46.7)	2 (13.4%)
$25,000–$49,999	13 (43.3%)	6 (40.0%)	7 (46.6%)
$50,000 or greater	3 (10.0%)	0 (0.0%)	3 (20.0%)
I choose not to answer	1 (3.3%)	1 (6.7%)	0 (0.0%)
Work/school, *N* (%)			
Full time/part time work	21 (70.0%)	10 (66.7%)	11 (73.3%)
School	3 (10.0%)	2 (13.3%)	1 (6.7%)
Other	6 (20.0%)	3 (20.0%)	3 (20.0%)

#### Semi-structured interviews

The semi-structured interviews encouraged discussions regarding experiences during the pandemic and the vaccine stance(s) of individuals within the Haitian community. Separate interview guides were created for community members and stakeholders to assess their thoughts. We obtained data regarding 9 main topics: (1) Thoughts/concerns about the pandemic, (2) Thoughts about COVID-19 vaccines, (3) Healthcare access during the study period, (4) Intrapersonal relationship dynamics, (5) Thoughts about individuals diagnosed with the COVID-19, (6) Prevention measures (e.g., wearing masks, hand hygiene, social distancing, and COVID-19 vaccination), (7) Mental health struggles and coping, (8) Food insecurity, and (9) Overall experiences during the COVID-19 pandemic.

The members of the community were asked questions about their own thoughts on the coronavirus pandemic, how it affected their personal lives, and whether they would take the vaccine(s) (which, at that time, were under development and beginning their initial release). The stakeholders were asked to relay their thoughts about the pandemic and the experiences of Haitian and Haitian American community members. Interview guides with verbatim questions are presented in Tables 2, 3.

**Table 2:** Qualitative interview guide for community stakeholders.

Topics	Questions	Quotes
Thoughts/concerns about COVID-19	How do you Haitians/Haitian Americans think or feel about the coronavirus pandemic? Please explain. Do you think Haitians/Haitian Americans have any concerns regarding the coronavirus? If so, what are the concerns and why?	*Personally, to me it’s a tragic thing that is happening. Everyone is worried about it, and everyone is wondering if eventually they will end up getting it*. *The whole fact they do not know what it is. The whole fact some of their friends started getting affected by it. Like they have lost somebody close to them or anything like that. And at first, they thought, “just drink some tea and it’ll help.” There were some people who were drinking tea 24/7 and still got infected by the disease. So that scares them away. It’s the unknown*.
Vaccine thoughts	Do you think the Haitians/Haitian Americans would take the vaccine?	*It’s all about medication so they are kind of scared about having to get a vaccine*. *If they are Haitian, they stay away from the vaccine*. *Yea and [there’s] not enough data, all the data. So yes of course, not that they cannot change, the fact that there’s not enough data, they are not up for it, no*.
Healthcare access	Do you think Haitians/Haitian Americans have had any problems accessing healthcare due to the coronavirus pandemic? If so, how so? Why? How do you think Haitians/Haitian Americans feel about going to see the doctor or going to the hospital at this time? Tell me more.	*Like I said, a lot of them do not have health coverage. It’s either their jobs aren’t providing it to them or they do not have enough money to have the proper insurance. And let us just say they find something super cheap, some of them say their covered until they go to the hospital where they are not covered. It scares them to death*. *They’re very scared. No one wants to go to the hospital. They feel like you go to the hospital and go get the corona*.
Intrapersonal relationship dynamics	How do you think the coronavirus has impacted Haitians/Haitian Americans’ interactions with family and friends? If so, how?	*Well from everywhere you could go to see your friends before And Haitians love muah (kiss sound), they huggy huggy, they kissy kissy. People cannot really do that now. When they came here, you have to A) try to give your hand first. They will hug. “I do not care about COVID. I want to give you a hug.” COVID affecting them because they love to give you hug, they love to give you kisses. Now they cannot deliver it.*
Thoughts on those with COVID-19	How do you think Haitians/Haitian Americans view people with the coronavirus?	*They will not talk to you, they do not even want you to say hi- do not look at them. But they care, they are not negative about it.*
Prevention measures	What do you think Haitians/Haitian Americans think about the current measures to prevent the spread of coronavirus such as wearing masks, using hand sanitizer etc.? What do you think Haitians/Haitian Americans are doing to protect themselves and others from the spread of coronavirus?	*laughs* yes…they…use… Uh hand sanitizer, they wear their mask under their nose *chuckles* most of them. So yes I do not think they are as afraid of it as the general population. Of course, there are exceptions but I’m always reminding them to cover their nose and you know the children are not covered. *It’s the teas. The natural remedies, um of course handwashing. I feel like that’s part of the culture, they make sure to wash their hands, sanitizer in their bag and their purse. You know, all these people walking in my store Haitians and last Spanish are the ones that will pull a bottle of hand sanitizer out of their purse.*
Mental health/coping	How do you think Haitians/Haitian Americans’ mental health has been during the coronavirus pandemic? Do you think Haitians/Haitian Americans have been doing anything to cope with the current coronavirus? If so, what?	*You could- in general, I notice more anxiety and that’s what I tell them. Some of them will come and say “I’m having palpitations, I’m feeling, I cannot sleep.” A lot of my patients are reporting insomnia, and I tell them “of course” look at what we living, the situation we live in.* *Talk to their friends, that’s it…. And pray. That’s the culture really.*
Food insecurity	Do you think the coronavirus pandemic has impacted Haitians/Haitian Americans’ ability to feed themselves and their families?	*At the same time, they did not have no money. (pause) But thanks God people survived. This the reason when we see, we start in the covid times start giving food to people. I remember one day someone call and say “I just came from the nursing home, I do not have since yesterday, I have not eat anything. I’m hungry.” It was by May when the covid just hit. “I do not have no food.” I say… my sister answer the phone I say, “Tell her you are going to bring her food” They say, “I do not have food.”*
Overall experiences during the COVID-19 pandemic	Do you think the coronavirus pandemic affected Haitians/Haitian Americans’ lives (for example, housing employment, access to medication etc.)? Other areas of their lives?	*Yeah, housing yes. If they do not have no apartment, no job. We have a housing problem. People lose they house, they could not go to work. And today I talk to someone I was so sad. So, she was paying her house $700, she lost—she could not have a job. She looking for a job right now she do a physical for it. She lost her house for $700 she could not got because she was sick, she did not go to work. They came out of the house, she lost the house. Now she’s renting from someone one, a room for $700.*

**Table 3:** Qualitative interview guide for community members.

Topics	Questions	Quotes
Thoughts/concerns about COVID-19	What do you think or feel about the coronavirus pandemic? Please explain. Do you have any concerns regarding the coronavirus? If so, what are the concerns and why?	*Corona is an unexpected situation, made us turn our life around. We all had to re-organize our day-to-day activities. Life suddenly had a whole different meaning, reality had to be acknowledged whether family wise, work wise and social wise. Life had to be put in perspective.*
Vaccine thoughts	Would you take the vaccine?	*A vaccine requires serious studies and tryouts before exposing it to the grand population. I have to many doubts about it and no I would not take it.* *I’m not about the vaccine. Because I’m afraid of the United States, this system. When I find a little Haitian doctor who speaks English, Spanish, or Creole, I feel better. But if you give me another doctor, I’m afraid. Because the doctors here, all they know is money.* *If the vaccine could help and everybody is cure…. It could be good.*
Healthcare access	Have you had any problems accessing healthcare due to the coronavirus pandemic? Tell me more. If so, how so? Why? How do you feel about going to see the doctor or going to the hospital at this time? Tell me more.	*Well, I used to work. When I used to work, I used to pay my insurance $115. But if you do not work, you cannot pay.* *Yes, it’s more difficult because you need an appointment for anything you need to do.* *Uncomfortable, many acquaintances and close friends and hospital workers confirmed contracting the virus at the hospital. So, I do not trust hospitals’ ways of dealing with the management of their facilities decontamination.*
Intrapersonal relationship dynamics	Has the coronavirus impacted your interactions with family and friends? If so, how?	*What really scares me is that everyone has to be distanced from one another, you cannot talk to someone’s face because you do not know if they are sick, you know? So, you have to stay away from everyone. You have to keep your distance.*
Thoughts on those with COVID-19	How do you view people with coronavirus 2019?	*Not differently than if I had the influenza. It’s all a matter of understanding that it can happen to anyone.* *I am very afraid of them…. Because I do not want to get sick. I feel bad they caught it… but very afraid (shaking head).*
Prevention measures	What do you think about the current measures to prevent the spread of coronavirus such as wearing masks, using hand sanitizer etc.? What are you doing to protect yourself and others from the spread of coronavirus?	*We just pray and drink tea.* *Well, I mean, I follow the CDC guidelines. I wash my hands several times a day. I put my mask. I keep my distance.* *Like me I wear my mask, every time I get in my car I apply the think on my hand, I use the hand sanitizer. That’s not enough for me, I do not feel as it’s enough you get it. But we are under God’s protection, we’ll keep on praying and thanking him.*
Mental health/coping	How has your mental health been during the coronavirus pandemic? Have you been doing anything to cope with the current coronavirus? If so, what?	*No too good, I feel I have no control…… (head bow down) no control of my life anymore.* *Yes, we pray a lot. We pray a lot and worship a lot. That’s what we doing.* *Well, I spent a lot of time watching with my grandkids. I watch a lot movies. I read books I keep my appointments with my primary care these other things I have been doing to make sure that mental health is alright.*
Food insecurity	Has the coronavirus pandemic impacted your ability to feed yourself and your family?	*Well, for example you have to work to be able to eat. I have a kid in Haiti, and I have a house in Haiti, and I have to pay it. But if you do not work, you cannot do anything. Even here (the US), you have to work to pay where you leave, when you sleep and to eat. If you do not work, you have nothing.* *I lost my job, and it is hard now…. To feed my family….*
Overall experiences during the COVID-19 pandemic	Has the coronavirus pandemic affected your life (for example, housing employment, access to medication etc.)? Other areas of your life?	*I did not have problems finding medication when COVID happened. My problem is I could not find jobs when I did not have a job, I did not have health insurance, that was my only problem, but they took care of me even without insurance.* *I have to wake up every day… in a world… I do not know anymore…. Everybody wearing a mask…. Every day in computer at home…. Every day is scared. My husband works still but it… is very hard to pay bills of the house…. I clean house when I can, and this help pay the food but it still not enough.*

#### Data analysis

A codebook was developed by the principal investigator, supervising researcher, and a study team member based on 10 interviews. A sample of 3 interviews was coded by both the principal investigator and a study team member using NVivo. The Kappas were above 0.8. The resulting codebook was used to code the remainder of the interviews. With the 30 interviews, we were able to achieve data saturation.

## Results

Table 1 shows the socio-demographic characteristics of the participants. Of the 30 participants, 15 were community members, and 15 were stakeholders. Participants self-identified as female (15; 50%) and male (14; 46.7%), while one preferred not to answer (1; 3.3%). The average age of the community members was 47.9 (SD = 14.27), while the stakeholders’ average was 39.5 (SD = 10.4). All 15 of the community members were born in Haiti. Of the stakeholders, 6 were born in the U.S., 8 were born in Haiti, and 1 was born elsewhere. Approximately 60% of the community members had not completed high school while 93% of the stakeholders graduated from high school or higher. Of the 30 participants, 86.7% identified as Christian. Over half (66.7%) of the community members and stakeholders reported that they were renting a house or apartment. More than 53% of the community members reported a household income of less than $25,000, and about 67% of them were working full- or part-time. Over half (66.7%) of the stakeholders reported an income of $25,000 or more, with 73.3% working full- or part-time. The community stakeholders represented community-based organizations, were medical professionals, or worked for the government. Twenty interviews were conducted in English and ten were in Haitian Creole.

Both community members and stakeholders provided insightful information regarding (1) thoughts/concerns about the pandemic, (2) thoughts about COVID-19 vaccines, (3) healthcare access during the study period, (4) intrapersonal relationship dynamics, (5) thoughts about individuals diagnosed with the COVID-19, (6) prevention measures (e.g., wearing masks, hand hygiene, social distancing, and COVID-19 vaccination), (7) mental health struggles and coping, (8) food insecurity, and (9) overall experiences during the COVID-19 pandemic.

### Thoughts/concerns regarding COVID-19

Overall, participants thought the COVID-19 had a negative effect on the Haitian community. Among this group, COVID-19 concern had been at the center of the participants’ thoughts due to misinformation about the disease, fear of contracting the infection, and absence of a cure. One participant noted: “*Personally, to me, it’s a tragic thing that is happening. Everyone is worried about it, and everyone is wondering if eventually they will end up getting it.*”

### Thoughts about vaccines

While 33% of the community members expressed that they would be willing to take the new COVID-19 vaccine, 67% responded that they would not take it or were hesitant. There were some concerns regarding the speed of the vaccine’s development and mistrust of the medical community. There were also suggestions of a conspiracy involving the COVID-19 vaccine. One participant suggested that “*If they are Haitian, they stay away from the vaccine*.” This suggests that culture may play a role.

### Healthcare access

The pandemic affected access to healthcare for our study participants. Many discussed losing their jobs and therefore not being able to pay for care. In addition, some clinics were closed, leading to worsened healthcare access. Both health services and employment/income, key social determinants, were affected. One of the stakeholders noted: “*Like I said, a lot of them do not have health coverage. It’s either their jobs aren’t providing it to them, or they do not have enough money to have the proper insurance. And let us just say they find something super cheap, some of them say they are covered until they go to the hospital where they are not covered. It scares them to death*.”

### Intrapersonal relationship dynamics

Overall, the COVID-19 pandemic had a negative effect on social relationships and environments. Some members of the community (3 out of 15 community members) experienced isolation and were unable to express themselves in a culturally congruent manner. One participant noted that “*You could go to see your friends before, and Haitians love muah (kissing sound), they huggy huggy, they kissy kissy. People cannot really do that now.*”

### Thoughts on those diagnosed

At that time, there was both concern for those diagnosed and some fear of being exposed to COVID. In addition, social stigma against individuals with COVID was not unusual. Social environments and social support networks were affected. All of the community members who were infected with COVID (3 out of 3 community members) expressed that they had felt isolated primarily because of the behavior of others towards them: *“They will not talk to you, they do not even want you to say hi- do not look at them. But they care, they are not negative about it.*”

### Prevention measures

Overall, it appeared that at that moment, the community was attempting to follow behavioral prevention measures. One participant noted that there might be a generational difference in willingness to follow guidelines, such that older individuals were more adherent. In addition, some participants (8 out of 15 community members) turned to more traditional prevention measures, arguing that herbal remedies could play a significant role in prevention. “*It’s the teas. The natural remedies, um of course handwashing. I feel like that’s part of the culture, they make sure to wash their hands, sanitizer in their bag and their purse.*”

### Mental health/coping

Prayer and spirituality was particularly important for many individuals (6 out of 15 community members) in the Haitian community for both coping and prevention. The participants of this study believed that religion and social support from friends played a healing role in coping with the COVID-19 pandemic. “*Talk to their friends, that’s it…. And pray. That’s the culture really.*”

### Food insecurity

Food insecurity was a problem for some (5 out of 15 community members). The participants connected job loss with an inability to provide essentials for their families such as food. “*Tell her you are going to bring her food. They say I do not have food.*”

### Overall experiences during the COVID-19 pandemic

COVID-19 appeared to affect members of the Haitian community on many levels. Employment was key. Several members reported that they lost their jobs or had a family member lose a job (7 out of 15 community members). Employment affected other areas of life as well, such as healthcare access and food insecurity. “*I mean it affects the Black community a lot because a lot of us was already into poverty so let us say a lot of people was losing their jobs and the government was not really helping us really. So, they were giving out stimulus but they not really doing anything to help the Black community with their needs.*”

## Discussion

In the U.S., the COVID-19 pandemic disproportionately impacted racial and ethnic minority groups negatively, including Haitians/Haitian Americans (2). Our findings agree with others suggesting that this population experiences similar barriers to healthcare as other immigrant communities in South FL (4). The sociodemographic characteristics of the participants in this study provide a holistic representation of this segment of the Miami community. With nearly three-fourths of the participants having been born in Haiti, the findings of this study offer unique insights that reflect the lived experiences and perceptions of first-generation immigrants, an essential factor when examining their responses to a global health crisis like the COVID-19 pandemic.

One key observation from this study is the prevailing apprehension towards the COVID-19 vaccine. The hesitancy mirrored findings from Chery et al., which also noted reluctance among the Haitian population due to factors such as misinformation, cultural beliefs, and lack of trust in the health system (12). The concerns regarding the rapid development of the COVID-19 vaccine and conspiracy theories seen in this sample suggest that external narratives play a significant role in shaping perceptions of vaccines. Such apprehensions are consistent with the community’s historical marginalization and the resulting mistrust towards medical interventions and historically based mistrust of the medical establishment, which can be linked to earlier health crises (15). Furthermore, studies from the Caribbean have found rural dwelling, lower education, and financial insecurity to be associated with vaccine hesitancy among Haitians/Haitian Americans (4, 16, 17). These barriers prompted Haitian/Haitian Americans to be less likely to be vaccinated against COVID-19 than other racial and ethnic minority groups (16–21). Avoidance of vaccines was also attributed to participants’ preference for traditional, cultural remedies over the use of Western medicine (19, 22–24). The apprehension towards the COVID-19 vaccine observed among Haitians/Haitian Americans parallels concerns seen in other minoritized populations within the US, such as Cubans, other Afro-Caribbean groups, and Latin Americans. These groups similarly grapple with misinformation, cultural beliefs, and a deep-seated mistrust towards the health system. For example, research among Latin American immigrants has highlighted significant hesitancy towards vaccinations, attributed to a combination of misinformation spread through social media and historical distrust in government and healthcare institutions (25–27). Similarly, studies on Black Americans have documented longstanding mistrust in the U.S. healthcare system, which has contributed to vaccine hesitancy, partly stemming from unethical historical medical experiments like the Tuskegee Syphilis Study (28). These patterns underscore a broader theme of mistrust among minoritized populations, stemming from historical injustices and compounded by current systemic barriers in healthcare access and information dissemination.

Our findings on food insecurity draw attention to the cascading effects of economic challenges. The interplay between employment, healthcare access, and food insecurity elucidated by study results is reminiscent of the multifaceted vulnerabilities, such as having jobs affected by economic downturns and healthcare or immigration barriers observed during past crises in the Haitian diaspora (29). The compounded vulnerabilities of Haitians/Haitian Americans are not isolated experiences but are shared across various minoritized communities. For instance, Afro-Caribbean and Latin American immigrants often encounter language barriers, immigration status concerns, and lack of culturally competent care, which exacerbate disparities in healthcare (30). Food insecurity, amplified by the pandemic, has similarly affected Black, Latino, and immigrant households at disproportionately high rates, reflecting broader systemic inequities (31, 32).

This study reveals structural challenges in urban settings like Miami, aligning with literature that delves into the challenges faced by immigrant communities in accessing healthcare (12, 33). The effects of the pandemic on intrapersonal relationships in the community suggest a deeper socio-cultural rift. Cultural congruence in expressing emotions and maintaining relationships is integral to communities with close-knit family and friendship ties, such as those of this community (34, 35). Thus, the reported isolation due to COVID-19 could have potential long-term implications on the community’s mental and emotional well-being.

Preventive measures used by this community reflected an interplay of traditional practices and global health guidelines. The significant role of herbal remedies signifies a blend of ancestral knowledge with contemporary health advice, similar to patterns seen in other minority communities (36). This blending was further echoed in the reliance on prayer and spirituality, aligning with past literature on the Haitian community’s coping mechanisms (37).

While the COVID-19 pandemic has affected communities globally, this study confirms the disproportionate negative experiences faced by members of the Haitian community in Miami, reflecting both universal challenges and unique socio-cultural dynamics. Although the COVID-19 pandemic started over 3 years ago, these data are important for addressing the support needs of this community and for overcoming vaccine hesitancy in future pandemics. Further studies are needed to explore these and other negative health consequences that disproportionately affected this population during COVID and other public health emergencies.

Although those who were interviewed brought up mistrust of the medical community, we were unable to distinguish between mistrust of the medical community and mistrust directed towards the public health community. Future studies should delineate and characterize these differing forms or reasons for mistrust directed at each of these two professional communities.

Finally, to address social determinants of health, structural interventions are needed to improve employment opportunities and other resources for this population to cope with the negative experiences due to the COVID-19 pandemic. Reducing healthcare barriers, building trust within the community, and improving transparency and communication are among the critical actions that can be taken to improve this population’s access and uptake of public health interventions like the COVID-19 vaccines. Addressing these challenges will require an integrated coordinated approach, taking into account the Haitian/Haitian American community’s cultural norms, historical experiences, and structural barriers. All of these lessons learned have practice implications for how medicine and public health should improve to better deal with the COVID-19 crisis and other similar issues that are likely to emerge or reemerge in future public health emergencies. Future work may benefit from comparing the experiences of Haitians and other communities using larger samples. Furthermore, future work on the Haitian community may benefit from *a priori* use of methodologies such as the Health Equity Impact Assessment.

## Data availability statement

The raw data supporting the conclusions of this article will be made available by the authors, without undue reservation.

## Ethics statement

The studies involving humans were approved by the University of Miami Institutional Review Board (Study #: 192 20200190). The studies were conducted in accordance with the local legislation and institutional requirements. Written informed consent from the [patients/participants OR patients/participants legal guardian/next of kin] was not required to participate in this study in accordance with the national legislation and the institutional requirements. Verbal consent from participants was approved by the University of Miami Institutional Review Board.

## Author contributions

CS: Conceptualization, Data curation, Formal analysis, Funding acquisition, Investigation, Project administration, Writing – original draft, Writing – review & editing. DR: Data curation, Formal analysis, Writing – original draft, Writing – review & editing. VE: Data curation, Writing – original draft, Writing – review & editing. MC: Writing – original draft, Writing – review & editing. MB: Writing – original draft, Writing – review & editing. DM: Writing – review & editing. AF: Writing – review & editing. MT: Writing – review & editing. AM: Writing – review & editing. MA: Conceptualization, Methodology, Supervision, Writing – review & editing. SD: Conceptualization, Methodology, Supervision, Writing – review & editing.
